# Making remote measurement technology work in multiple sclerosis, epilepsy and depression: survey of healthcare professionals

**DOI:** 10.1186/s12911-022-01856-z

**Published:** 2022-05-07

**Authors:** J. A. Andrews, M. P. Craven, A. R. Lang, B. Guo, R. Morriss, C. Hollis, The RADAR-CNS Consortium

**Affiliations:** 1 NIHR MindTech MedTech Co-operative, Institute of Mental Health, University of Nottingham, Nottingham, UK; 2 Mental Health and Clinical Neurosciences, School of Medicine, University of Nottingham, Nottingham, UK; 3 Human Factors Research Group, Faculty of Engineering, University of Nottingham, Nottingham, UK; 4 NIHR Nottingham Biomedical Research Centre, University of Nottingham, Nottingham, UK; 5 ARC-EM, School of Medicine, University of Nottingham, Nottingham, UK; 6 Kings College London, London, UK

**Keywords:** Remote measurement technology, Multiple sclerosis, Epilepsy, Depression, Healthcare professionals, Survey

## Abstract

**Background:**

Epilepsy, multiple sclerosis (MS) and depression are long term, central nervous system disorders which have a significant impact on everyday life. Evaluating symptoms of these conditions is problematic and typically involves repeated visits to a clinic. Remote measurement technology (RMT), consisting of smartphone apps and wearables, may offer a way to improve upon existing methods of managing these conditions. The present study aimed to establish the practical requirements that would enable clinical integration of data from patients’ RMT, according to healthcare professionals.

**Methods:**

This paper reports findings from an online survey of 1006 healthcare professionals currently working in the care of people with epilepsy, MS or depression. The survey included questions on types of data considered useful, how often data should be collected, the value of RMT data, preferred methods of accessing the data, benefits and challenges to RMT implementation, impact of RMT data on clinical practice, and requirement for technical support. The survey was presented on the JISC online surveys platform.

**Results:**

Among this sample of 1006 healthcare professionals, respondents were positive about the benefits of RMT, with 73.2% indicating their service would be likely or highly likely to benefit from the implementation of RMT in patient care plans. The data from patients’ RMT devices should be made available to all nursing and medical team members and could be reviewed between consultations where flagged by the system. However, results suggest it is also likely that RMT data would be reviewed in preparation for and during a consultation with a patient. Time to review information is likely to be one of the greatest barriers to successful implementation of RMT in clinical practice.

**Conclusions:**

While further work would be required to quantify the benefits of RMT in clinical practice, the findings from this survey suggest that a wide array of clinical team members treating epilepsy, MS and depression would find benefit from RMT data in the care of their patients. Findings presented could inform the implementation of RMT and other digital interventions in the clinical management of a range of neurological and mental health conditions.

**Supplementary Information:**

The online version contains supplementary material available at 10.1186/s12911-022-01856-z.

## Background

Epilepsy, multiple sclerosis (MS) and depression are long term conditions which have a significant impact on the everyday lives of patients who live with them. Epilepsy is a common neurological condition [1] characterised by repeated seizures which may or may not have a motor component. People with epilepsy are at risk of sudden unexpected death in epilepsy (SUDEP), which has an incidence of 1.16 deaths per thousand people with epilepsy [2]. MS is a neurological condition in which the brain is affected by plaques, which appear in conjunction with ‘attacks’ which cause deterioration in cognitive and physical functioning [3]. MS has a relapsing–remitting form and a progressive form, both of which have a significant impact on the lives of those who live with them. Depression is the most common mental illness [4] and is characterised by periods of low mood, low motivation, anhedonia, excessive/insufficient sleep, poor/excessive appetite, and tiredness [5].

The management of all three of these central nervous system disorders typically involves repeated contact with healthcare professionals (HCPs), in which the patient’s symptoms are evaluated and any treatment they are receiving is reviewed. However, the evaluation of patients’ symptoms is problematic in each of these conditions. In epilepsy, there is poor accuracy of patient-provided seizure records [6], while measures for depression require patients to recall their mental states over long time periods although their recall may be affected by present mental state [7]. In MS, some patients struggle to evidence changes in their condition that they have noticed in the standard tests offered at clinic. Depression is common in MS, though it is frequently undetected [8].

Remote measurement technology (RMT) may offer a way to improve upon existing methods of managing these three conditions. RMT consists of digital technologies (apps and wearables) used to record active (user-inputted) and passive (auto-recorded) data which may provide information about a patient’s condition. Research has begun to explore the potential of RMT in applications to mood monitoring in depression and bipolar disorder [9–11], seizure detection in epilepsy [12, 13] and functional assessment of multiple sclerosis via smartphone apps [14].

Over 6 years, the RADAR-CNS (Remote Assessment of Disease and Relapse – Central Nervous System) project seeks to explore the feasibility of the use of RMT in the care of patients with epilepsy, MS or depression. In addition to three, large-scale observational studies using RMT to collect passive and active data from patients with each of the conditions in home (MS, epilepsy, depression) and hospital (epilepsy) settings [15–17], the project has involved studies to understand the perspectives of HCPs on ways these technologies could fit into clinical pathways [18, 19]. This paper builds on a prior interview study within the same project where HCP interviewees indicated multiple types of data that could be collected using RMT which would be useful in the management of patients with epilepsy, MS or depression [18]. Findings also showed that RMT data would be useful at specific times, including: initial referral into the care pathway; after any changes in treatment; and prior to routine appointments once the condition was stable. Interviewees considered it important that primary care teams, specialist nurses and all clinical, but not administrative, members of a team, should have access to RMT data from patients, preferably via the electronic patient record. They also indicated that it would be essential for clinicians to have knowledge of the margins of error of any predictions or measures made using RMT data.

Other prior research exploring clinicians’ experiences of RMT has shown that RMT data can enable clinicians to delegate tasks to suitably qualified but more junior staff, reducing their own workload [20, 21]. However, in the specific case of epilepsy, healthcare professionals have suggested that RMT may in fact increase workload and may cause increased patient anxiety [22]. In the area of mental health, clinicians have raised concerns about the reliability and accuracy of sleep and mood tracking apps, although they recognise their potential in facilitating the collection of routine data [23]. Clinicians treating multiple sclerosis have commented on the potential to collect more in-depth data from patients using wearable devices than can be collected in clinic [24]. However, it has been proposed that a number of paradigm shifts will be required for clinicians to integrate remote measurement technology into their work, including the set-up of virtual offices from which clinicians can operate, and a shift toward continuity of data exchange and connection between patients and their clinical team [25]. Despite this need for important changes in the way clinicians work to implement RMT, no research to date has explored at scale the views of healthcare professionals on the practical requirements for, and value of, introducing RMT in central nervous system disorders.

This paper reports the findings of an online survey of healthcare professionals (HCPs) exploring views on the potential application of RMT in clinical pathways for the management of MS, epilepsy and depression in Europe. The aim of the study was to understand the potential value of RMT data in clinical pathways, and the practical requirements for its implementation, including potential barriers to use, from the perspective of healthcare professionals in Europe. Initial findings from the first half of this survey have been published [26], including that use of smartphones for clinical purposes is widespread among clinicians (60%), that the majority of respondents (76%) had experience of treating patients who use RMT, and that data from these devices has an impact on their clinical practice. This paper reports the findings from the second part of the survey, setting out the types of data considered useful in each condition, the potential for benefit, the points at which HCPs can envisage using the data, as well as perceived requirements for training and potential challenges to implementation. These are considered in the context of clinical pathways for the care of epilepsy, MS and depression in Europe.

## Methods

### Design

Survey methodology was considered appropriate to source data at scale from a range of HCPs working in the care of people with epilepsy, MS or depression. Surveys are useful to allow clinicians to provide their views without spending too long doing so and to allow them to take part at a time of their choosing [27]. The survey was presented on the JISC Online Surveys platform [28]. The survey instrument consisted of 26 questions, including both short, free text response items and closed questions. The full survey is provided in Additional file 1. Ethical approval was granted by the University of Nottingham Research Ethics Committee (ref 277-1802).

Demographic information was collected on age, clinical specialism, job role, clinical setting and country of work. There were seven further sections to the survey, covering: current use of digital services and devices; using digital devices for long term monitoring; the value of RMT; access to and use of data; technical support requirements; and closing remarks, for final additional comments. The selection of questions included in the survey was informed by expert elicitation, in which clinical academic members of the project consortium were invited to suggest areas where gathering the opinions of a large number of HCPs would be useful.

Recruitment was conducted via the East Midlands Clinical Research Network, and by inviting the RADAR-CNS research consortium to pass details of the study on to their clinical colleagues. The Clinical Research Network advertised the study to all healthcare trusts across the United Kingdom. Twenty trusts contacted the research team about the study, and 17 of these advertised the survey to their staff. The survey was also disseminated via official online social media accounts, via the consortium website (www.radar-cns.org) and in project newsletters. The original target set for recruitment was 100 responses, with a minimum of 30 respondents per condition. Within each condition, we sought to recruit 10 medical staff, 10 nursing staff and 10 members of the wider multidisciplinary team. As we expected only to use descriptive statistics to analyse the data, we did not calculate a sample size calculation. The first response was received on the 4th February 2019 and the last was received on the 30th March 2020. Respondents were required to complete the survey in a single sitting. Completion took around 15 min. Inclusion criteria were that the respondent was a healthcare professional and that they currently worked in the care of people with epilepsy, MS or depression.

### Analysis

Quantitative data were analysed using frequencies, percentages and chi-squared analyses. Confidence intervals for proportions were calculated at the 95% level. Qualitative results from free text boxes were analysed using content analysis to identify commonalities. The aim of collecting free text qualitative data was to allow respondents to provide additional descriptive detail or context to answers. Microsoft Excel, IBM SPSS 26 and Stata SE 16 were used for analysis and to generate graphs, with confidence intervals calculated in Stata SE 16 and chi square tests computed in IBM SPSS 26.

## Results

After removing 3 entries in data cleaning for insincere completion of the questionnaire or improper completion of the consent form, 1006 completed survey responses were analysed, greatly exceeding our target for recruitment. Table 1 presents demographic data from participants who responded to the survey. Job role and country were free text responses which were subsequently categorised. Age and clinical setting were discrete choice items, while specialism was a multi-select item, where respondents selected all that applied (Additional file 1). The majority of responses, 974/1006 (96.8%), were from clinicians working in the United Kingdom (UK). A broad range of ages were represented from age 18 upwards, with the greatest number (293/1006, 29.3%) in the 41–50 category and fewest (52/1006, 5.2%) in the 60+ category. A wide range of specialisms responded to the survey (respondents could select more than one), with greater representation from mental health roles (587/1006, 58.3%). Job roles were similarly diverse, with representation from all major job families. In terms of clinical setting, the least representation was from tertiary specialist care centres (54/1006, 5.4%), while the highest was from staff in mental health trust outpatients in secondary care (254/1006, 25.2%).

**Table 1:** Demographics of respondents to the survey

Category	n	%
*Age*		
18–30	161	16.0%
31–40	248	24.7%
41–50	293	29.1%
51–60	247	24.6%
60+	52	5.2%
No response	5	0.5%
*Specialism*		
Neurology	112	11.1%
Mood disorders	121	12.0%
Mental health	587	58.3%
Epilepsy	73	7.3%
Multiple sclerosis	55	5.5%
Depression	165	16.4%
General practice	152	15.1%
Psychology	126	12.5%
Social care	32	3.2%
Other	119	11.8%
*Job role*		
Allied Health Professionals	112	11.1%
Doctor (excl GP)	138	13.7%
GP	118	11.7%
Research/healthcare science	24	2.4%
Management	40	4.0%
Nursing	268	26.6%
Pharmacy	15	1.5%
Psychological professions	157	15.6%
Student	10	1.0%
Wider healthcare team	76	7.6%
Not clear	48	4.8%
*Clinical setting*		
Primary care/general practice	193	19.2%
Secondary care—hospital trust, inpatients	91	9.0%
Secondary care—hospital trust, outpatients	82	8.2%
Secondary care—mental health trust, inpatients	127	12.6%
Secondary care—mental health trust, outpatients	254	25.2%
Specialist tertiary care centre	54	5.4%
Community care	173	17.2%
Other	32	3.2%
*Country of employment*		
United Kingdom	974	96.8%
Portugal	21	2.1%
Belgium	2	0.2%
Italy	2	0.2%
Germany	1	0.1%
Ireland	1	0.1%
Israel	1	0.1%
Mexico	1	0.1%
Switzerland	1	0.1%
No response	2	0.2%

### Presentation of the results

Responses to the first part of the survey, questions 1–10, covered informed consent and respondents’ current use of RMT and apps in clinical practice. These results are presented elsewhere [26]. The remaining data from the main survey (from questions 11–24) are grouped into four general areas, ‘RMT data types of interest’, ‘Accessing RMT data in patient care’, ‘Benefits and challenges to patients using RMT’, and ‘Training required to make use of RMT’. These are each presented below.

### RMT data types of interest

Questions 11, 11a and 12 asked respondents to indicate whether they would consider certain types of data to be useful in the management of epilepsy, MS or depression and provided space for free text suggestions. Results from questions 11 and 12 showed that body movements, heart rate, environmental features, sleep quality, mood, concentration, attention and memory were considered useful to monitor across all three conditions. In addition, free text responses to question 11a suggested it could be beneficial to monitor exercise, nutrition (including alcohol consumption), anxiety, social contact and adherence to treatment regimes (including medication).

**Fig. 1:**
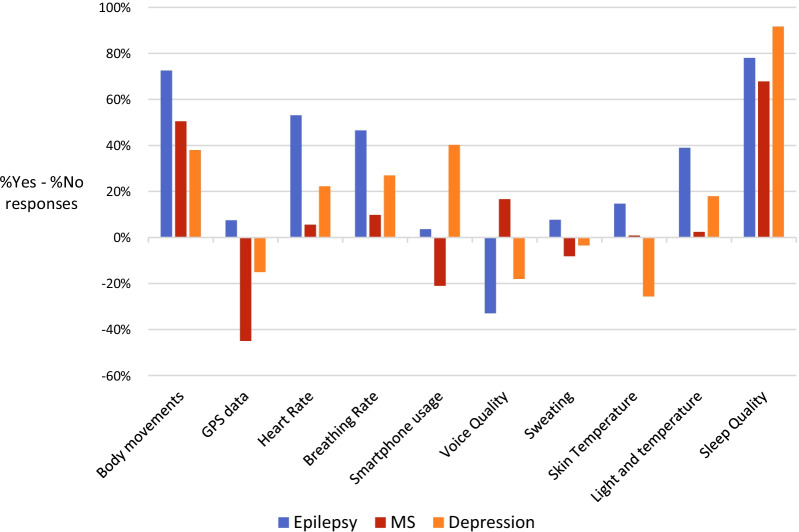
Respondents’ views on the usefulness of different (pre-selected) types of data that could be collected using RMT, across three different central nervous system disorders (Question 11). Percentages plotted here are results of subtracting percentage of ‘no’ responses from percentage of ‘yes’ responses in each data type and each condition. Thus bars above the x axis show items with a greater number of ‘yes’ responses, while bars below the x axis show items with a greater number of ‘no’ responses

Some data types were considered helpful only in one or two of the conditions (Fig. 1). Further to the data types deemed useful in all conditions, respondents in epilepsy considered it useful to monitor breathing rate, sweating and skin temperature. In multiple sclerosis specific data types considered useful were breathing rate, voice quality and skin temperature. In depression, clinicians considered it important to measure smartphone usage.

**Fig. 2:**
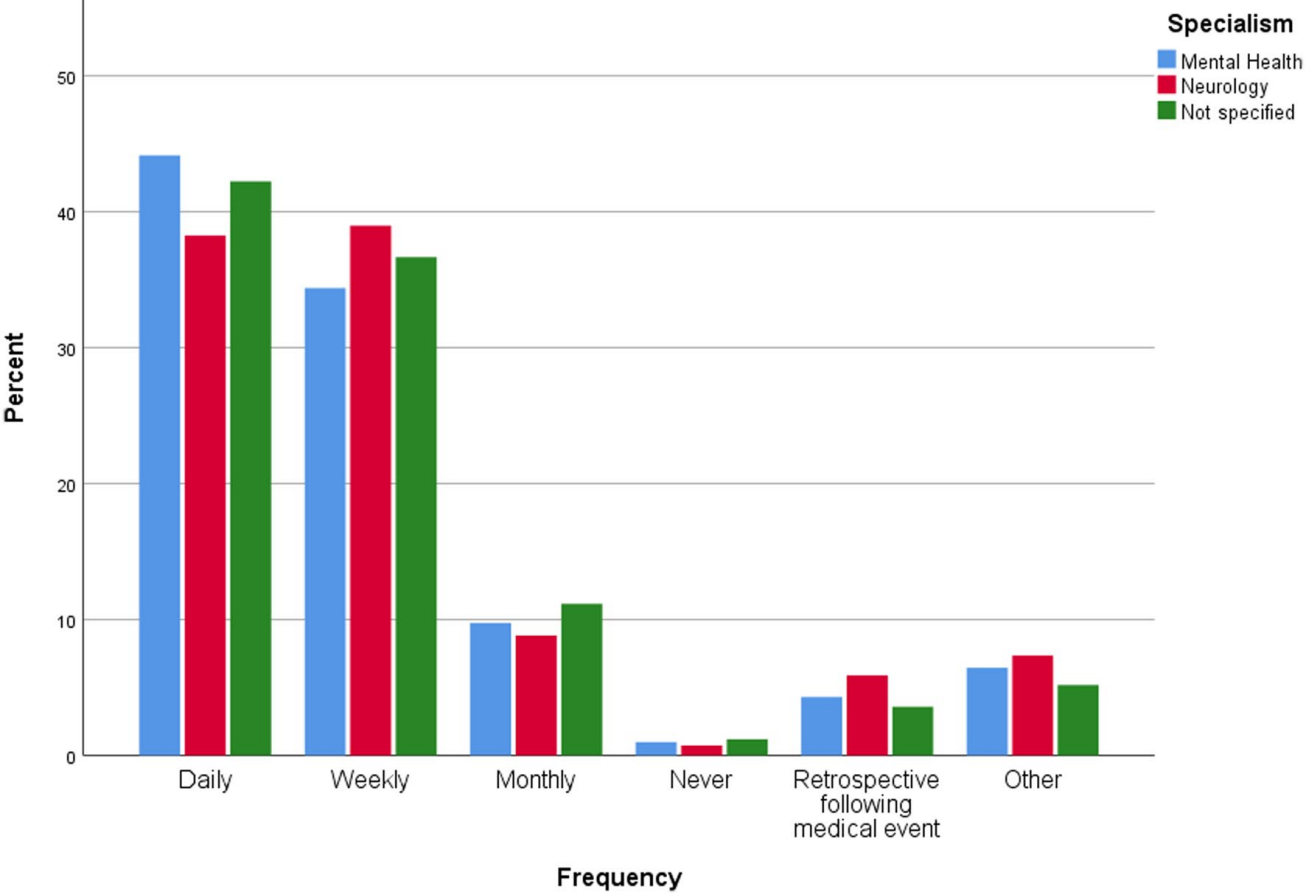
Frequency of mood reports considered useful (Question 13). Chart shows percentages of each (summarised) specialism selecting each answer

Question 13 asked respondents to indicate how often it would be useful to collect self-reported mood data from patients. Figure 2 shows how responses to this question varied according to specialism. The majority of responses indicated that daily or weekly mood reports would be most preferable. Among mental health professionals, a greater percentage of respondents indicated that daily mood reports would be useful compared to weekly reports. The same pattern was not observed among neurology specialists.

### Accessing RMT data in patient care

Questions 14, 16, 17 and 18 asked respondents to consider who in their clinical team would access RMT data (Q14), when in a patient’s care the data would be most useful (Qs 16 & 17), and how clinicians would prefer to access the data (Q18). Table 2 shows data from question 14, regarding members of a clinical team who would make use of RMT data. Nurses were considered best placed to benefit from the data, although it was considered by 116/1006 (11.5%) of respondents that all medical staff members would be able to benefit.

**Table 2:** Responses to question 14, a free text question on clinical team members considered able to benefit from RMT data in their work. Similar responses were grouped, and groups with greater than a threshold of 10 occurrences in the data were included in this table

Free text response with > 10 responses	n
‘Nurses’/‘Nursing and medical staff’	214
‘Medical’/‘Medics’/‘Medical team’/‘Medical staff’	116
‘All’/‘Everyone’	100
‘Me’/‘Myself’/‘Ourselves’	99
‘GPs’/‘General Practitioners’/‘Primary Care’	53
‘Clinical staff’/‘Clinical team’/‘Clinicians’	30
‘Secondary care’	23
‘Specialist doctor’/‘Specialist nurse’/‘Specialist team’	15
‘Therapists’	14
‘Don’t know’/‘Unsure’	13
‘Psychologists’	11

In response to Question 16, the majority of those responding confirmed that there were specific decision points relying on mood-based variables (599/946, 63.3%) and specific decision points relying on relapse/seizure measures (495/857, 57.8%) where remote measurement data would be useful in their clinical practice. Respondents were less confident of the benefits from remote measurement data in informing decision points that were not specific to relapse or that relied on contextual factors.

Results from question 17 showed that a majority of respondents considered it likely or highly likely that they would access RMT data at each of the given times in the question (in preparation for a consultation with a patient (568/978, 58.1%), during a consultation with a patient (602/982, 61.3%), in between consultations if the system were to flag up data/reasons for concern (583/970, 60.1%), and post medical event e.g. seizure and/or relapse (620/949, 65.3%)). The highest proportion of ‘highly likely’ responses was recorded for reviewing data following a medical event such as a seizure (267/949, 28.1%), indicating that this was the occasion when RMT data would be considered most useful. Question 18 asked participants to rank four pre-determined ways of accessing RMT data. The results showed a similar level of preference assigned to all four suggested ways of accessing the data, with mean averages of rankings for all four options falling between 1.9 and 2.5 (1: most preferred to 4: least preferred).

### Benefits and challenges to patients using RMT

**Fig. 3:**
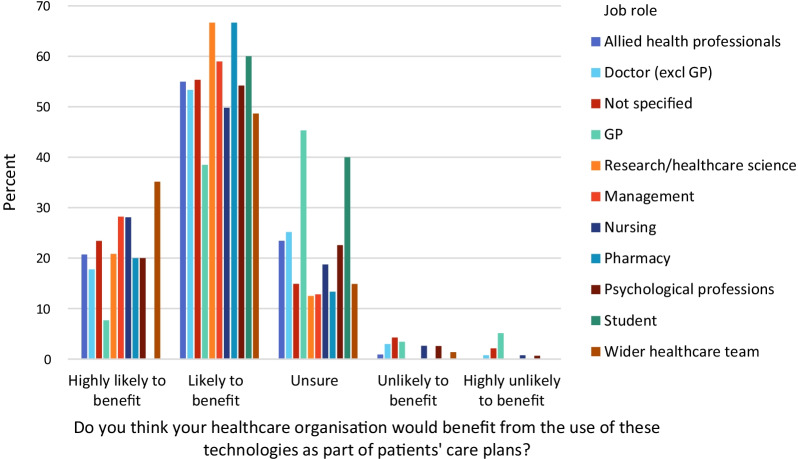
Respondent views on whether their healthcare organisation would be likely to benefit from the implementation of remote measurement technologies as part of patients’ care plans (Question 15), separated by job type

Question 15 asked respondents to indicate whether they considered the use of RMT in patients’ care plans would benefit their healthcare organisation. On a 5-item response scale from ‘highly likely to benefit’ to ‘highly unlikely to benefit’, 511/994 item respondents (50.8%) indicated their service would be ‘likely to benefit’, with 217/994 (21.6%) considering it ‘highly likely’. A chi squared test showed that there were significant differences between job roles (χ^2^ = 92.452, p < 0.001). Figure 3 shows a breakdown of these results by job role. GPs and medical students were most likely to indicate they were ‘unsure’ whether use of RMT would benefit their healthcare organisation. GPs were also most likely to indicate it would be ‘highly unlikely’ the organisation would benefit, although this was a small proportion of respondents (n = 6/117, 5%).

**Fig. 4:**
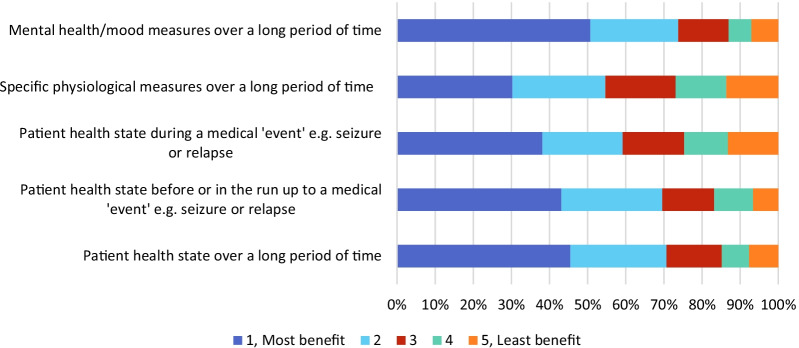
Respondent rankings of how beneficial different types of information from remote measurement technology are likely to be in clinical practice (Question 19), from greatest perceived benefit (1, dark blue) to least (5, orange)

Question 19 asked respondents to rank different types of information potentially available from RMT in terms of their potential to benefit clinical practice. Analysis of the rankings showed the greatest perceived benefit to derive from ‘Information about mental health/mood measures over a long period of time’, with least benefit perceived in ‘Information about specific physiological measures over a long period of time’ (Fig. 4). Free text responses indicated that RMT would also have the benefit of increasing patients’ engagement in their own care, and thereby improve self-management.

**Fig. 5:**
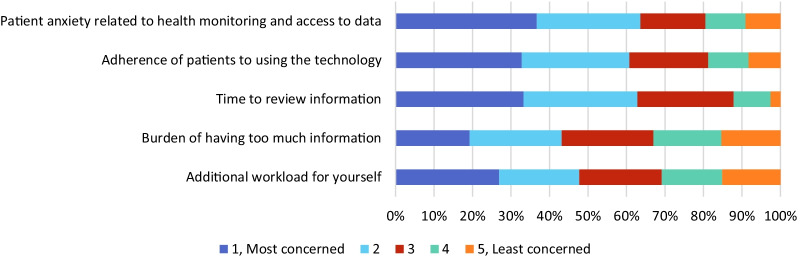
Respondent rankings of barriers related to the implementation of RMT (Question 20). Respondents ranked each from 1 to 5 where 1 indicated most concern and 5 least

Question 20 asked respondents to rank barriers associated with RMT implementation (Fig. 5). Overall, ‘time to review information’ and ‘patient anxiety related to health monitoring and access to data’ were seen as the greatest barriers associated with RMT implementation (Fig. 5). Free text responses supplemented these pre-selected items with other barriers to the implementation of RMT, which included reference to: accessibility of the technology; issues around confidentiality and data protection; patient honesty and reliability to ensure consistent use rather than use only when symptoms were more severe; patients not understanding the technology or being well enough informed to make sense of the data; and, in epilepsy specifically, that some seizure types may be more difficult to detect.

**Fig. 6:**
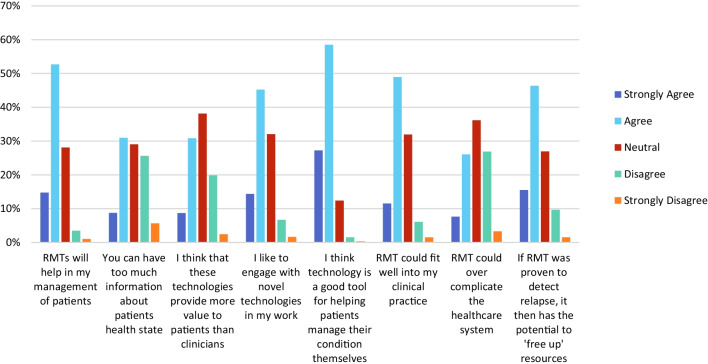
Respondent opinions of the potential usefulness of patient use of RMT to monitor a condition (Question 21)

A generally positive view of the overall potential of RMT was found in responses to question 21. Some concerns were also clear, as 386/972 (39.7%) of item respondents indicated agreement or strong agreement with the statement that ‘*you can have too much information about patients’ health state*’ (Fig. 6), however this was lower than for other items including ‘*if RMT was proven to detect relapse, it has the potential to ‘free up’ resources*’ (602/973, 61.9%) and ‘*RMTs will help in my management of patients*’ (658/976, 67.4%). This indicates that overall, respondents were more positive about the potential of RMT than they were concerned about the volume of data it would generate (Fig. 6).

### Training requirements

**Fig. 7:**
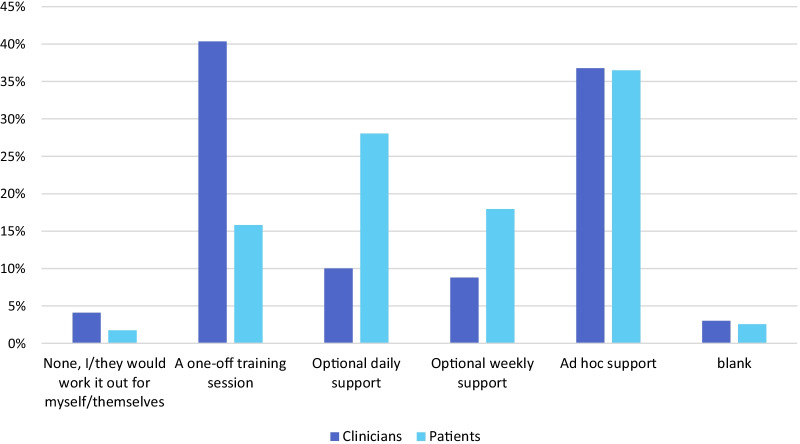
Amount of technical support considered by healthcare professionals to be necessary for healthcare professionals and their patients for the successful implementation of RMT (Questions 22 and 24)

**Fig. 8:**
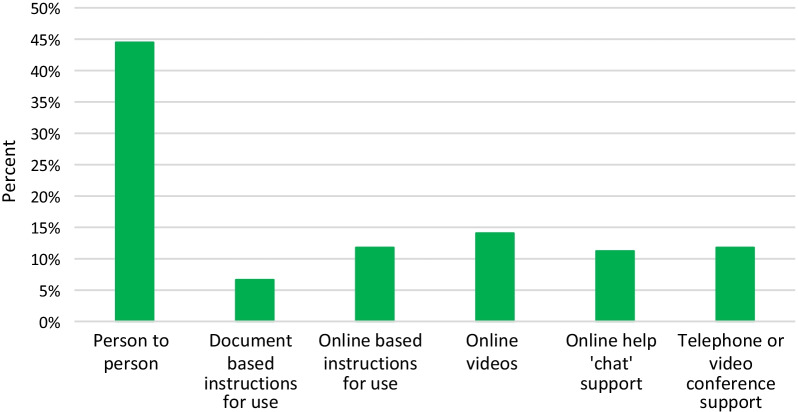
Mode of technical support preferred by respondents (Question 23). Bars represent percentage of item respondents ranking each mode of support as their highest preference

Questions 22–24 asked respondents to consider the technical support requirements that they and their patients would require to be able to make best use of RMT (Fig. 7). 395 of 979 item respondents (40.3%) indicated that clinicians would require a one-off training session while 360/979 (36.8%) selected that support should be provided on an ad hoc basis. With respect to the type of support that clinicians considered patients would require, the greatest percentage of item respondents (358/981, 36.5%) indicated that patients would require ad hoc support, with 275/981 (28.0%) suggesting optional daily support would be required (Fig. 7). The preferred means of receiving technical support for RMT was by person-to-person support (Fig. 8). This was ranked highest by 638/979 respondents (65.2%).

## Discussion

### Principal findings

This sample of survey respondents were positive about the benefits of RMT, with 73.2% (CI 70.4–76.0%) considering that their service would be likely or highly likely to benefit from implementing RMT in patient care plans. This supports prior work in this area, including our own, which has shown that clinicians are in general positively disposed toward remote measurement technology [18, 29]. The results from this survey have added to our previous work [18, 19, 26] by providing an indication of healthcare professionals’ views on how RMT could work in practice, based on a large sample. While the data from patients’ RMT devices could be reviewed between consultations if the system flagged reasons for concern, the results suggest it is also likely that RMT data would be reviewed in preparation for, and during, a consultation with a patient, or for a patient to self-manage their own condition. In relation to data access, healthcare professionals would prefer to access RMT data during consultation via the electronic patient record, indicating a requirement for patients’ data to be centrally held via a secure portal. Respondents indicated that the data collected should be made available to nursing staff and all medical team members . Time to review the information, patient adherence, and potential for increased patient anxiety are the greatest barriers to the successful implementation of RMT.

Analysis of the rankings in question 19 showed the greatest perceived benefit to derive from ‘Information about mental health/mood measures over a long period of time’, with least benefit perceived in ‘Information about physiological measures over a long period of time’, which may be expected given the relatively larger proportion of staff working in mental health care rather than neurology in the res pondent demographics.

Among the conditions covered by the survey (epilepsy, MS and depression), our survey showed sleep measurements, actigraphy (body movements), heart rate, breathing rate and measurements of ambient light and temperature to be considered useful by clinicians treating all three of the conditions. There was more variability between conditions for other measures including GPS data, skin temperature, sweating, smartphone usage and voice quality. The low rating of voice quality in depression was surprising given a growing amount of research interest in this area [30]. It may be that clinicians consider this research to be at a nascent stage or are otherwise unaware of existing tools for its measurement and analysis. For multiple sclerosis, GPS data was not considered to be helpful, perhaps because typical diagnostic indicators of MS progression, including timed walking tests, rely on measurements of gait, where actigraphy is likely to provide a greater level of resolution [31]. Data on smartphone usage was regarded as most useful in depression, which is supported by cross-sectional survey studies reporting correlates between depression symptoms and reported smartphone usage and addiction [32–34]. Free text responses supplemented these findings, with data on nutrition highlighted as potentially useful information across all three conditions. These findings could inform feature selection for future predictive models for use in these conditions.

Among the job roles of respondents, it was surprising to find that a large proportion of GP respondents (53/117, 45.3%) were ‘unsure’ that their healthcare organisation would benefit if RMT was used in patient care plans, particularly given that they were more likely than other health care professionals to recognise an impact on their job role from RMT data [26]. This may be because primary care is mostly local and responsive to health events as they happen, in contrast to secondary care where a strategic, longer-term view can be taken. The value of RMT may be greatest where hospital and specialist community teams (secondary and tertiary care) can use RMT as an alternative to hospital admission, specifying the most useful measurements to be taken for a patient, recording baselines, and analysing trends over time. The role of RMT may be more limited in primary care where most care involves the assessment of a patient’s immediate health presentation.

Training has been shown to increase clinicians’ familiarity with, and willingness to use, technologies in their work [35, 36]. The need for training in the use of RMT for both clinicians and patients was recognised by survey respondents. Either a one-off training session or ad hoc support would be preferable above other forms of training for HCPs to make use of RMT data and the preference would be for this to be delivered person-to-person. Authors of prior work suggest that the provision of training seminars and workshops might have a positive influence on attitudes towards healthcare technologies, since these could increase familiarity and experience with some technologies and thereby increase acceptance [37]. The present study adds to these findings by indicating the amount of training support that clinicians consider they would need in relation to RMT (one-off training/ad hoc support) and highlighting their preference for person-to-person support over instruction manuals, online videos and other media-based support.

One of the highest ranked concern s regarding the implementation of RMT in clinical practice in our study was patient adherence to use of the technology. Recent work has highlighted patients’ own concerns with regard to adhering to RMT measurement regimes, for example the bulkiness and visibility of a device meaning it could be stigmatizing [38], and the fact that responding to momentary sampling questionnaires may not fit with their lifestyle or other commitments [39]. In addition, our data show that where patients have access to their data, this may cause them anxiety, indicating a need for careful design of dashboards/apps reporting data back to patients.

Presentation of data to clinicians is similarly important, with time to review data also ranked as an important concern. Our survey explored the times that a clinician would be interested in reviewing the data from patients’ RMT, with a preference shown for accessing the data following a medical event such as a seizure. We cannot tell from these survey data what the desired level of granularity may be in the presentation of such data to the clinician. Actigraphy data is known to be complex to organise in a useful way for clinicians, with clinicians in different clinical disciplines requiring different forms of data presentation and differing levels of detail [40]. A need has been recognised to allow clinicians to focus on subsets of patient-generated data, as well as identify overall trends and present new data alongside contextual data [41]. The responses here may assume that data review requirements for both clinicians and patients will be achieved but RMT systems and corresponding dashboards may need significant development to meet these requirements.

### Implications for practice

Findings from this study suggest that health care professionals are likely to be interested in using RMT in the care of patients living with MS, depression or epilepsy, once the safety, usability and cost effectiveness of these technologies have been evaluated. While some barriers to use were recognised as important to respondents in this study, our findings would suggest that clinicians remain positive about the value that RMT can offer. Findings suggest there may be less buy-in from primary care in relation to RMT and use cases for this setting will therefore need to be more clearly established. We additionally highlight clinicians’ preference for in-person training in relation to new technologies, which may be informative for those working in medical education.

In our study, RMT was recognised as a useful tool for helping patients to self-manage their condition, in addition to benefits in clinical patient management and monitoring. Self-management interventions for depression are known to be effective in improving patients’ levels of self-efficacy and in increasing self-management behaviours [42]. However, using RMT for patient self-management is not perceived by clinicians to be a suitable replacement for face-to-face care [18], and prior work has emphasised the need for discussion between patients and clinicians regarding remotely collected data for the effective use of self-management tools [43]. Thus, self-management via RMT may be seen to offer an additional benefit for patients rather than replacing any elements of face-to-face care. The benefits of this additional offer will need to be evaluated for cost effectiveness, incorporating the requirement for ad hoc/optional daily technical support for patients using RMT for self-management, as recognised by survey respondents.

### Limitations

Although this survey was completed by over 1,000 healthcare professionals, it is possible there was a sampling bias toward those who have a more positive view of technology, as respondents were self-selecting. In addition, despite attempts to recruit from European countries outside the UK, there was little representation from countries other than the UK among respondents. However, respondents’ demographics showed a large range of job roles and age ranges, indicating a broad sample in these regards. We did not collect data on gender in this survey, although it may have been interesting to explore differences in views on this basis.

While the majority of questions in the survey did not specifically mention the three conditions under consideration, the external validity of the survey may be low with respect to other conditions, as the design was intended to focus only on multiple sclerosis, epilepsy and depression. However, the study results have supported and added to the findings from our prior interviews of healthcare professionals in the same clinical areas. One further limitation is that the survey was conducted prior to the COVID-19 pandemic, which may affect the reliability of the results post-pandemic. In many countries, healthcare professionals may have utilised RMT data more frequently in the absence of usual face to face clinics during lockdown restrictions. Views on the value of RMT and on its potential for implementation may have been modified through this further experience.

## Conclusions

The findings from this survey suggest that a wide array of clinical team members treating epilepsy, MS and depression would benefit from RMT data from their patients, although the types of data deemed useful, and the frequency at which these should be collected, vary by condition. The main barriers to overcome prior to implementation are patient adherence, potential for increased patient anxiety and clinical time required to review the information. These findings provide a clear sense of the practical requirements for making use of RMT data in clinical practice from the perspective of healthcare professionals while also highlighting barriers where further development work is required. Further work could include comparison studies to determine the qualities of wearable devices that make them more likely to see continued usage, and explorations of how passive data could be used as a proxy for more attention-demanding active reporting tasks. Good precision in such applications of RMT may offer improved methods for collecting data from patients which is more complete and potentially also more granular. Further work in the form of pilot studies and RCTs (or alternative designs) is also required to evaluate the clinical and cost effectiveness of implementing RMT for the collection of outcome measure data in the three conditions covered here.

## Supplementary Information

**Additional file 1**. Survey instrument, presented to respondents using the JISC online surveys platform (www.onlinesurveys.ac.uk).

## Data Availability

The datasets used and/or analysed during the current study are not publicly available due to agreements within the research consortium but are available from the corresponding author on reasonable request and with permission of the RADAR-CNS consortium.

## Abbreviations

COVID-19: Coronavirus Disease 2019
GP: General Practitioner
HCP: Healthcare Professional
MS: Multiple Sclerosis
NHS: National Health Service
NIHR: National Institute for Health Research
RADAR-CNS: Remote Assessment of Disease and Relapse—Central Nervous System
RMT: Remote Measurement Technology
SUDEP: Sudden Unexpected Death in Epilepsy
UK: United Kingdom
